# The National Pension Fund and the Registration of Nurses

**Published:** 1888-01-07

**Authors:** 


### THE NURSING MIRROR.

Communications for this column should be addressed The Mirror, care of the Editor, 38, Old Jewry, London, E.C.

WE have had pleasure in acceding to the request of several correspondents by commencing this new department of THE HOSPITAL. We have always tried to meet the requirements of a large number of readers by doing justice to the nursing profession. Experience has shown, however, that it is desirable to devote a certain space to nurses and nursing each week, and this want will now be fully met. The nurse occupies a recognised position in the system of every hospital, as she does in every household where sickness has to be treated. Hence, all that affects the welfare of the nurse intimately concerns the managers of these institutions, and the heads of households throughout the country. Items of news, calculated to interest nurses, will always be welcome for this column.

This week we commence with a letter from the lady superintendent of a provincial Nursing Home. We publish this statement because the *bonâ fides* of the writer is beyond question, although she shows an amount of misapprehension and confusion on the question she refers to not easily to be accounted for. It is desirable that all should have a precise knowledge of the actual facts and circumstances connected with these two schemes, with which object we have taken some trouble to ascertain them, and to set them forth in detail point by point at the end of our correspondent's statement, which runs as follows :—

"The National Pension Fund and the Registration of Nures. A scheme, organised by Mr. H. C. Burdett, entitled 'The National Pension Fund for Trained Nurses,' has now been for some time before the public. No one who is in any degree acquainted with trained nurses and their work can fail to acknowledge that the establishment of such a fund would be in the highest degree beneficial and desirable. In THE HOSPITAL, a weekly paper devoted to the consideration of medical, nursing, and sanitary matters, Mr. Burdett has from time to time expounded the lines on which he thinks this 'National Pension Fund' should be established; and in the main, his propositions appear to be laid down on a sound and financial basis. The point at which issue must be joined with Mr. Burdett, is upon 'the *Registration* of Trained Nurses.' According to Mr. Burdett's scheme, the eligibility of a pensioner under 'the National Pension Fund' is to be determined by *Registration*. In the 'Rules for the Registration of Trained Nurses,' issued by The Hospitals Association (to which the National Pension Fund is affiliated), it is provided that 'a nurse seeking to be placed on the register, must furnish satisfactory proof that she has worked for *at least one year* on the staff of a hospital or infirmary.' Now this arbitrary rule at once shuts the door of the so-called '*National*' Pension Fund upon a class of trained nurses, second to none in the nation for devotion, skill, intelligence, and usefulness, viz., the trained nurses forming the staff of *private* nursing institutions. Few of these excellent women have 'worked for at least one year on the staff of a hospital or infirmary.' They have for the greater part been sent by the heads of their institutions to be trained in London or other hospitals for periods varying from six to eight or nine *months*—amply sufficient to fit them for entrance upon the duties of private nursing. Indeed, those who from experience are best qualified to speak on this subject are aware that a longer period of hospital training is unnecessary and often detrimental to the private nurse, whose subsequent mode of life and work is widely different from that of the regular hospital nurse. An experience of sixteen years, spent in close daily contact with private nurses, enables me to say without hesitation that their life is a more arduous and anxious one, both bodily and mentally, than that of hospital nurses can possibly be. All honour to those noble women who spend their lives in the wards of our great London and provincial hospitals, nursing the sick and dying, tending them through terrible operations, and watching over them in the delirium of infectious fevers and diseases. Let no one think that I would disparage this splendid work or those devoted workers. But equally noble and devoted are the women on whose behelf I write, but whom Mr. Burdett, if his registration scheme be adopted, practically places outside the benefit of the National Pension Fund. There may be some people who think that the strain upon these two classes of nurses is equally great, but even if this were the case, which I do not for one moment admit, the injustice of providing a pension for the one class whilst practically ignoring the claims of the other marks a glaring defect in Mr. Burdett's scheme. 'The National Pension Fund for Trained Nurses' is a wide and far-reaching title; let it be organised on a wide and far-reaching basis worthy of its catholic appellation. Then might we fittingly appeal to our Most Gracious Queen to build it up from her Jubilee offering on a substantial and wide foundation."

